# A Decade of Liver Transplantation in the United States: Drivers of Discard and Underutilization

**DOI:** 10.1097/TXD.0000000000001605

**Published:** 2024-05-03

**Authors:** Julia Torabi, Rachel Todd, L. Leonie van Leeuwen, Yuki Bekki, Matthew Holzner, Jang Moon, Tom Schiano, Sander S. Florman, Mohammed Zeeshan Akhtar

**Affiliations:** 1 Department of General Surgery, Mount Sinai Hospital, New York, NY.; 2 Icahn School of Medicine at Mount Sinai, New York, NY.; 3 Recanati/Miller Transplantation Institute, Mount Sinai Hospital, New York, NY.

## Abstract

**Background:**

Organ shortage remains a major challenge for the field of transplantation. Maximizing utilization and minimizing discard of available organs is crucial to reduce waitlist times. Our aim was to investigate the landscape of liver recovery, discard over the past decade in the United States, and identify areas to reduce organ discard.

**Methods:**

This study used the Scientific Registry of Transplant Recipients United Network for Organ Sharing database to analyze the rates and associated reasons of discarded organs from 2010 to 2021. All deceased donors were evaluated, and data were analyzed by organ type, year, and region. Organ disposition was analyzed by year and region. Donor demographics and liver biopsy data were also analyzed.

**Results:**

The volume of liver transplantation increased steadily, with a 44% increase from 2010 to 2021. Donation after circulatory death transplantation increased by 239%, comprising 10.6% of transplants in 2021, yet discard rates remained high at 30% for this donor subset. For all donor types, the liver discard rate has remained stable around 10% despite a 74% increase in available donors. Seventy percent of liver discards were attributed to organ factors, with biopsy findings accounting for 40% of all discards. Of livers that were biopsied, 70% had macrosteatosis of <30%.

**Conclusions:**

Analysis of trends in transplantation and discard allow for identifying areas of underutilization. Donation after circulatory death livers have expanded the pool of transplanted livers but remain discarded at high rates. Significant differences remain in discard rates between geographic regions. We identify several areas to lower the discard rates. The expanding role of machine perfusion may allow for utilization of previously discarded organs.

The organ shortage continues to be one of the major challenges in abdominal transplantation. In the United States, mortality on the waitlist approaches 25% for those without exception points. At the end of 2020, 12.1% of liver patients have remained on the waitlist for >5 y.^1^ In Europe, for comparison, the wait time for organs is much lower.^2^ Given the lifesaving and disease-altering nature of liver transplantation, the lack of availability of good-quality organs translates to loss of significant numbers of life years, and the need to address the organ deficit remains one of the greatest challenges in transplantation.

Over the last decade, concerted efforts have been made to mitigate the organ deficit. These include expansion of the deceased donor pool via use of older donors, hepatitis C-positive donors, and increasingly utilizing donors declared dead based on cardiovascular criteria, or donation after circulatory death (DCD).^3-6^

The allocation policy has also been reformed repeatedly in attempts to maximize the sharing potential and to recover organs for those patients who need them the most.^7,8^ Other innovative advancements such as organ preservation with hypothermic and normothermic perfusion pumps and normothermic regional perfusion (NRP) of DCD donors offer promising potential to reshape the field of transplant.^9,10^

Areas for consideration in addressing the organ deficit include understanding what proportion of currently discarded organs are being underutilized, that is, either discarded after procurement or not procured at all, and considering what proportion of these organs could potentially be salvaged and utilized for transplant. While there are clear indications for discarding organs, such as malignancy, many factors contribute to the complex decision-making process as to whether to accept or reject an organ for an individual. The advent of modern technologies, biomarkers, logistical flexibility, and further insights into biology of organ injury could allow for safely increasing utilization of discarded organs.

We sought to understand the national landscape of liver recovery and discard over the last 11 y. Our goal was to reexamine drivers of discard and nonutilization, using this as a basis to evaluate the success of interventions made and to identify areas for development. We sought to understand how discard and underutilization were related to several factors, including donor type and the underlying factors that led to organ discard and donor underutilization.

## MATERIALS AND METHODS

To perform this study, we used data from the Scientific Registry of Transplant Recipients data. This database includes information on all US donors, candidates on the waitlist, and those who have received transplants submitted by members of the Organ Procurement and Transplantation Network. Our analysis included all data from the “Deceased Donor” file from January 1, 2010, to December 1, 2021.

Using the “Deceased Donor” file, data were extracted for all donors and analyzed by organ type. Data relevant to the following endpoints were extracted total number of donors, recovered organs, discarded organs, and transplanted organs. These were used to calculate discard rates by year. Donor factors such as donation after expanded criteria donor (ECD), DCD, Kidney Donor Profile Index, and biopsy findings were extracted. Nonrecovery and discard reasons were extracted using the database codes. For liver donors given the considerable number of discards coded as “other,” we used the variable “LI_DISCARD_CD_OSTXT” to reclassify the reason for discard. In the evaluation of ECDs, we reclassified donors as standard criteria donors, DCD, and ECD using the variables “ECD_DONOR” and “NON_HRT_DONOR.” Thus, ECD donors included all ECDs except for DCD donors.

Discard rate was the ratio of number of discarded livers to the number of recovered livers. Analysis was performed of the discard rate per region. Regions were grouped into high, medium, and low discard based on the following criteria. The range of discard rates were divided into tertiles (low: 4.7–8.5, moderate: 8.5–12.3, high: 12.3–16). To evaluate this further, we reviewed the ratio between organ discard and transplants performed per region as a comparator.

Statistical analysis was performed using IBM SPSS, Version 28.0.0.0. Graphs were generated using GraphPad Prism 9.3.1. This study was approved by the Institutional Review Board.

## RESULTS

### Liver Volumes, Sharing of Organs, Discard Rates, and Nonrecovery

**FIGURE 1.**
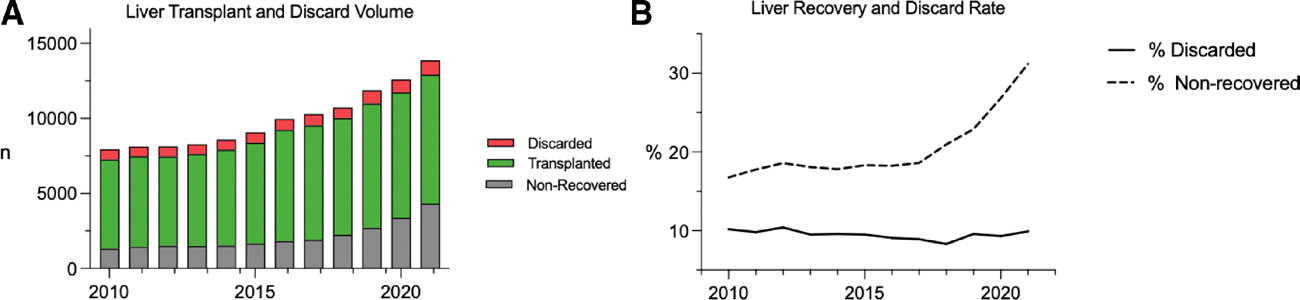
Liver transplant volume and discard rate from 2010 to 2021. A, All potential deceased donor livers and outcome of nonrecovery, discard, or transplantation from 2010 to 2021. B, The national nonrecovery rate and discard rate of recovered deceased donor liver allografts.

There was a 74% increase in available donors from 2010 to 2021 (7943–13 863 available donors), equating to an average increment of 538 donors per annum. From 2010 to 2021, the overall volume of liver transplantation increased by 44%. The percentage of livers from potential donors that went unrecovered increased from 17% to 31% over the 11-y period. Discard rates of recovered livers remained stable with a low of 8.3% in 2018 and a high of 10.4% in 2012 (Figure 1).

### Transplant and Discard Rates by Donor Type

**FIGURE 2.**
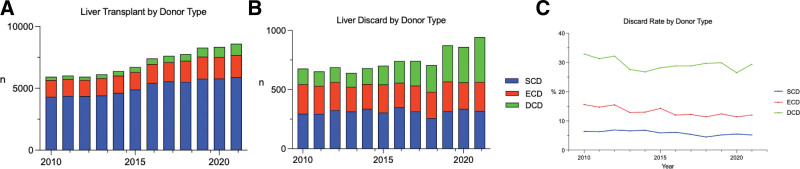
Discard and utilization of SCD, ECD, and DCD donors between 2010 and 2021. A, Transplanted livers by donor type from 2010 to 2021. B, Discarded livers by donor type between 2010 and 2021. C, Discard rate by donor type between 2010 and 2021. DCD, donation after circulatory death; ECD, expanded criteria donor; SCD, standard criteria donor.

Standard criteria and extended criteria DBD donors had modest increases of utilization of 36% and 32%, respectively, >11 y. From 2010 to 2021, transplantation of DCD donors increased by 239%, accounting for 10.6% of transplanted livers in 2021 compared with only 4.5% in 2010. Standard donor discard rates had a slight decrease from 6.4% to 5.2%. ECD donors discard rates decreased from 15.6% to 12%. Discard rates were highest for DCD donors, although, with a slight decrease from 32.9% in 2010 to 29.4% in 2021 (Figure 2).

**FIGURE 3.**
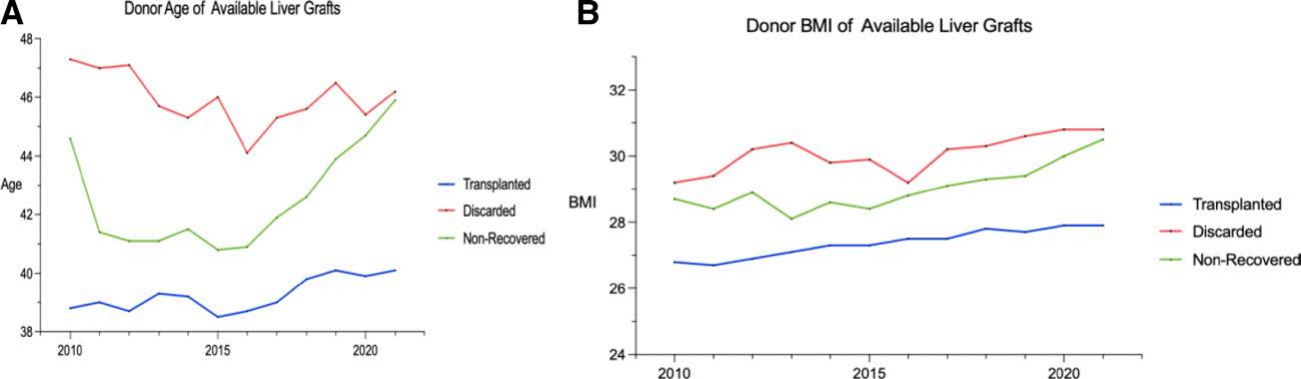
How age and BMI correlate with usability of organs for transplant. Donor (A) age and (B) BMI of transplanted, discard, and nonrecovered liver grafts between 2010 and 2021. BMI, body mass index.

The mean donor age of transplanted livers remained relatively static between 38 and 40 y old. For discarded and nonrecovered livers, the mean donor age was higher ranging from 44–47 y to 41–46 y, respectively (Figure 3A). Similarly, donor body mass index for transplanted livers remained static with a mean between 27 and 28 kg/m^2^. Discarded and nonrecovered liver donor body mass index trended slightly upward 29–31 (Figure 3B).

### Regional Trends in Discards Rates

**FIGURE 4.**
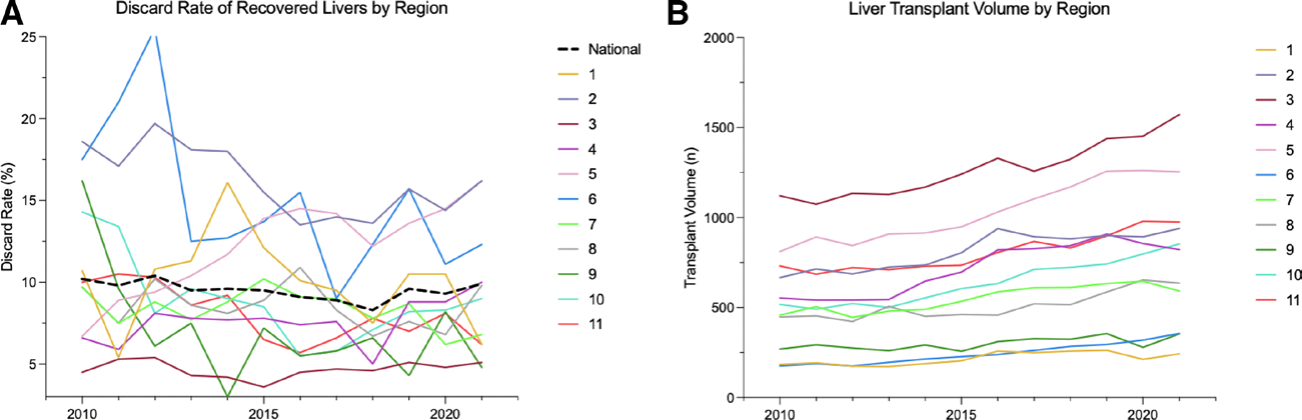
Discard rates and transplant volume between 2010 and 2021 by region.

Overall discard rates during the 11-y period varied substantially by region with the lowest rate of discard occurring in region 3 (4.7%) and the highest rate occurring in region 2 (16%) (Table 1). Within regions, there was yearly fluctuation of discard rates between 2010 and 2021 (Figure 4). Regions 3, 4, 7, 8, and 11 had relatively low variability in discard rate of <5% range of discard rates. While regions 1, 5, 6, and 9 had greater variability with range of discard rates >9%.

**TABLE 1.** Regional comparison of discard rates and ECD donor utilization

Regional discard rate tertile	Region	Discard rate (%)	Transplant volume (n/1000)	Range of yearly discard rate between 2010 and 2021 (%)	Transplant/discard ratio	Transplants imported from other region (%)	DCD transplants (%)	Transplanted grafts with steatosis >30% (%)
High	2	16.0	9.8	6.3	5.2	14.8	5.4	1.9
	6	14.5	2.9	16.5	5.9	15.3	10.0	0.8
	5	12.6	12.4	9.5	6.9	2.2	7.7	1.8
Moderate	1	10.0	2.6	10.7	9.0	10.2	5.7	2.6
	10	8.7	7.7	8.7	10.5	13.3	9.3	5
	8	8.5	6.1	4.2	10.7	15.3	6.8	3.6
	7	8.3	6.6	4.0	11.0	9.2	10	4.7
Low	11	7.9	9.7	4.7	11.6	19.1	5.2	5.6
	4	7.7	8.6	5.0	11.9	8.7	6.1	1.8
	9	7.0	3.6	13.2	13.3	13.8	5.2	4
	3	4.7	15.2	1.8	20.3	9.0	6.0	1.2

DCD, donation after circulatory dearth; ECD, expanded criteria donor.

Regions were grouped into tertiles of low, moderate and high discard. There was no correlation between discard rate and transplant volume within regions (Table 1). Nor was there any correlation between discard rates and percent of transplants imported from other regions (Table 1; Figure 4). Regional variation of DCD utilization ranged between 5.2% and 10.0%, with regions 6, 7, and 10 being highest utilizers of DCD donors (9.3%–10%).

### Factors Driving Organ Discard

The predominant reasons for discarding were organ factors (70%) and procurement factors (18%). The most common organ factor cited as reason for discard was biopsy findings, which accounted for 40% of all discards. Other discard reasons related to organ factors included diseased or unsuitable organ (17%), anatomic abnormalities (8%), and organ size (2%). Factors attributed to liver discard related to procurement factors included prolonged warm ischemic time (8.7%), organ trauma (3.5%), poor procurement flush (11.9%), and prolonged cold ischemic time (3.4%). Notably, 3.2% of livers were discarded due to recipient issues and were unable to be reallocated. No recipient was able to be located after the initial center declined in 5.5% of livers (Table 2).

**TABLE 2.** Factors attributed to liver discard after recovery or liver nonrecovery

Reasons for liver discard	n (%)	Reason for nonrecovery	n (%)
Donor factors	102 (1.1)	Donor factors	
Procurements factors		Donor history	1491 (5.6)
Organ trauma	312 (3.5)	Donor instability	1108 (4.2)
WIT	776 (8.7)	Organ factors	
CIT	275 (3.1)	Diseased organ	1239 (4.7)
Poor flush	194 (2.2)	OR evaluation	4169 (15.7)
Time constraints	32 (0.4)	Anatomic abnormalities	41 (0.2)
Donor instability	36 (0.4)	Poor organ function	2289 (8.6)
Organ factors		Biopsy findings	1585 (6.0)
Diseased organ	636 (7.1)	Procurement	
Visualization	643 (7.2)	Organ damage	193 (0.7)
Nonhepatic findings	220 (2.5)	Time constraints	1691 (6.4)
Size	172 (1.9)	Family/cultural	579 (2.2)
Biopsy findings	3511 (39.4)	Research	4824 (18.2)
Poor organ function	359 (4.0)	No recipient	1556 (5.9)
Anatomic abnormalities	711 (8.0)	Organ refused	2871 (10.8)
Recipient unsuitable	289 (3.2)	Other	2846 (10.7)
No recipient	491 (5.5)		
Other	145 (1.6)		

CIT, cold ischemic time; OR, operating room; WIT, warm ischemic time.

Over 11 y, the reasons for discarding remained stable, yet the rate of discards due to organ factors decreased from 73% to 61%. Discard due to biopsy findings decreased from 42% to 27%. Discard related to procurement factors increased from 17% to 22%. Discard secondary to lack of an identified recipient increased from 2.4% to 8.9%.

**FIGURE 5.**
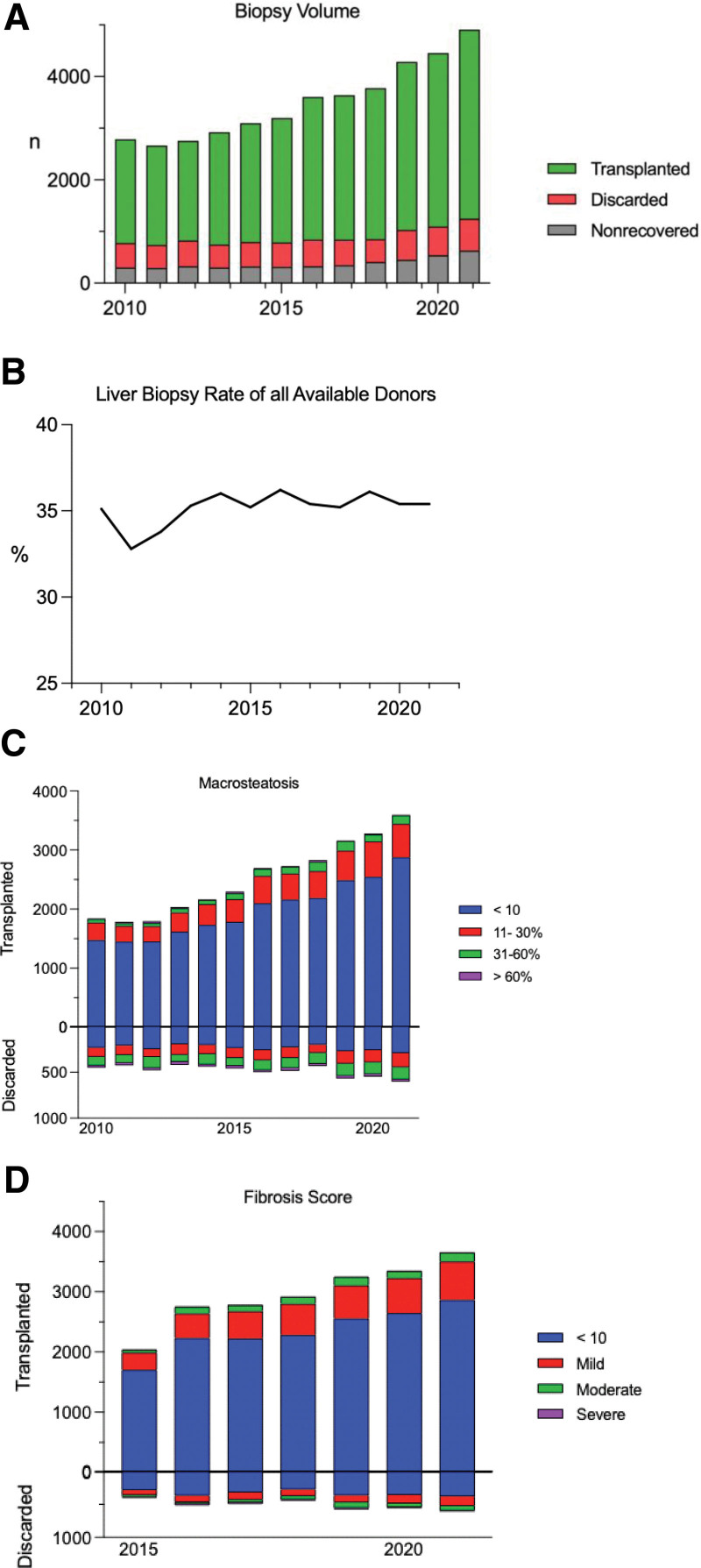
Liver biopsy volumes and rates from 2010 to 2021. A, Volume of liver biopsies performed of nonrecovered, discarded, and transplanted livers by year. B, Liver biopsy rate of all available donors by year. C, Macrosteatosis of biopsied livers. D, Fibrosis score of livers.

In this cohort, liver biopsy was performed in 23.9% of transplanted livers, 39.9% of discarded livers, and 11.3% of nonrecovered livers. From 2010 to 2021, the total number of biopsies increased by 57%. However, the rate of biopsy remained stable, ranging from a low of 32.8% in 2011 to a high of 36.2% in 2016 (Figure 5).

From 2010 to 2021, 95% of livers that were biopsied and subsequently transplanted had macrosteatosis of <30%. Livers that were biopsied and subsequently discarded with macrosteatosis <30% consisted of 72% of all discards. Discard percentage of livers with macrosteatosis <30% remained relatively stable with a low of 66.7% in 2018 and a high of 75% in 2015. Fibrosis scores of biopsied livers demonstrated a slight increase in utilization of mildly fibrotic livers from 14% to 17.5%, although these cases accounted for a small volume of transplanted livers (Figure 5).

### Factors Driving Nonrecovery of Livers

The most common reasons for not recovering a liver were attributed to organ factors (35.2%), which included unsuitable organs after being ruled out in operating room (15.7%), poor organ function (8.6%), or biopsy findings (6.0%). Donor factors including history or instability comprised 9.8% of nonrecovered livers. Other factors included time constraints (6.4%) or being unable to locate a recipient (16.7%). Livers used for research comprised 18.2% of nonrecovered livers.

From 2010 to 2021, the most common reason for nonrecovery was consistently organ factors, although the proportion of these cases decreased from 47% to 30%. Recipient factors increased from 4% to 9%, and organ refusal increased from 6.5% to 16%. Nonrecovery due to time constraints increased from 4.5% to 8%.

## DISCUSSION

In this study, we reviewed available Scientific Registry of Transplant Recipients data on deceased donor liver transplantation to gain an up-to-date understanding of how organ discard and donor nonutilization have changed in the United States over the last decade. Our aim was to identify windows of opportunities to decrease liver discard and underutilization of organ donors.

### DCD Donor Organs Are Still Highly Discarded and Underutilized

In the last decade, we have seen an increase in the volume of liver transplantation, predominantly driven by the increased utilization of DCD liver for transplantation supported by research suggestive acceptable outcomes for select donor-recipient pairs. Despite this increase, almost 30% of livers discarded are DCD donors. That said, the promotion of DCD donor transplants performed in the United States still lags far behind European countries.^11^

What drives the relative underutilization of DCD organs in the United States is multifaceted, but it is partly due to the healthcare model differences that make transplant institutions more accountable for complications or failures.^12^ In addition, the rules, regulations, and societal understanding of DCD donation are better established in Europe.^13^ With that said, DCD donation remains the largest source of potential underutilized donors and transplant centers are clearly turning to using livers from these donors as demonstrated by the growth of DCD liver transplants over the last 3 y. The aggressiveness of transplant centers to pursue livers from these donors should be championed, with acceptance that at least in the short-term this may result in decreased discard.

### Why Are Organs Discarded in the United States?

The reasons for liver discard are multifaceted. The predominant reason for discard is due to the biopsy findings and procurement factors, that is, prolonged warm ischemia time and injury. Pretransplant liver biopsy findings that typically result in the turning down of a liver include steatosis and fibrosis. Several studies have demonstrated an association between histological assessment and outcomes, although many of these associations are historical, and more recent data demonstrating these tools lack specificity.^14,15^ That said, histological assessment remains a valuable tool for the assessment of organs and to date remains one of the major assessment criteria for considering higher risk organs for transplant.

Our analysis demonstrates a stable biopsy rate from 2010 to 2021. While some data has suggested expanding the limits of steatotic graft utilization,^16^ our results demonstrate that the utilization of livers with steatosis >30% remained largely unchanged, with a high of 6.5% in 2018 and a low of 3.8% in 2014.

### What Factors Drive Underutilization of Donors in the United States?

Our analysis demonstrated that nonrecovery of organs from potential organ donors in the United States is driven by a multitude of reasons, the majority of which pertain to the nonsuitability of the donor organs for transplant purposes. Many of these factors are absolute contraindications to organ allocation, although some are continuing to be challenged as a reason for nonutilization. For example, HIV-positive donors to HIV recipient transplantation has been performed successfully by several centers throughout the United States. Although the data is not sufficiently granular to allow full extrapolation, we expect that part of the reasoning behind “no recipient” or “refused” reasons for nonutilization was owing to these risks. The extent of the nonutilization, however, is considerable, with 10 000 livers not being retrieved over a 10-y period. We advocate for this to be an area to focus research going forward.

### Geographic Disparities in Liver Discard Rates

We observed substantial variation between discard rates across regions. Geographic disparities are known to the transplant community and have been previously addressed by policy changes such as Share 35 and acuity circles. Interestingly, the regional discard rate did not correlate with transplant volume, importation rate, or DCD utilization. This suggests factors outside of those captured in this study drive this regional variation. However, these differences may offer additional target for decreasing discard rates. Certain regions may have higher tolerance for marginal organs that may be considered unacceptable in others. Current preservation strategies may limit the distances organs may travel thus necessitating discard.

### Reducing Discard and Nonutilization Using Machine Preservation

Our data suggests that there are several potential high-yield targets that would allow for improved abdominal organ utilization and reduction of discard rates. One promising area is the rise of machine perfusion, hypothermic machine perfusion, and normothermic machine perfusion (NMP), which allows for ex vivo preservation. Early trials have demonstrated the success of both techniques.^17-19^ While machine perfusion remains costly and time-consuming to set up, streamlining this process for wider use may have significant impacts on organ utilization.

The relatively short cold ischemic time accepted for liver transplant constrains the time and distance for liver sharing. In this study time constraints, cold ischemic time and donor-recipient mismatch comprised 14% of discards. If otherwise adequate, such grafts could be potentially transplanted if longer and oxygenated preservation was possible. Prolonged cold ischemic times using dual hypothermic oxygenated perfusion are currently explored in Europe.^20^ Portable cold devices would provide a great solution to the larger distances between donor and recipient hospital that are seen in the United States. Longer preservation times by implementing NMP would potentially allow for reallocation, translating to 100 liver grafts per year.

Another potential area for utilization of machine perfusion is in DCD donors, which are increasingly utilized but remain discarded at high rates. Dual hypothermic oxygenated perfusion has shown to decrease posttransplant complications in DCD liver transplantation, and NMP has shown to give a significant benefit in DCD donors with prolonged warm ischemic time.^9,18,21^

The use of NMP in steatosis livers also represents a substantial pool of nonutilized livers. Animal models and preliminary human trials suggest that NMP may protect against ischemia-reperfusion injury in steatotic livers. While a sizable percentage of steatotic livers may ultimately be unsuitable for transplantation, half of livers discarded due to biopsy findings being under the accepted macrosteatosis percentage of 30%. Furthermore, NMP could even be implemented as a therapeutic platform to treat organs with antisteatotic drugs.^22^ However, further research in this area is crucial as it represents a massive proportion of nonutilized livers, particularly given the rise of obesity in America and subsequent decline in liver graft quality.^23^

Another rising machine perfusion technique is NRP. In situ, the NRP of DCD donors converts the donor operation to a more standard donor operation type, thereby reducing the discard of organs due to procurement challenges and injuries. NRP also carries the benefit in resuscitating cellular function before transportation of an organ and has been shown by groups to reduce ischemic cholangiopathy rates in liver transplantation recipients.^21,24^

We observed trends in organ discards and utilization over the last decade. Utilization of DCD donors has compromised a large portion of the increased liver transplant volume, however, remain discarded at high rate. Discard rates vary substantially between geographic regions. Improvement in preservation techniques and sharing between regions can reduce discard of organs due to geographic and time constraints. Given the rise of obesity and decline in liver graft quality understanding organ discard and underutilization is critical to maximize use of recovered livers.

## References

[1] Kwong AJ Ebel NH Kim WR. OPTN/SRTR 2020 annual data report: liver. Am J Transplant. 2022;22(Suppl 2):204–309. 35266621 10.1111/ajt.16978

[2] Eurotransplant Annual Report. Statistics Report Library. Available at https://statistics.eurotransplant.org. Accessed August 19, 2023.

[3] Halazun KJ Quillin RC Rosenblatt R. Expanding the margins: high volume utilization of marginal liver grafts among >2000 liver transplants at a single institution. Ann Surg. 2017;266:441–449. 28657945 10.1097/SLA.0000000000002383

[4] Danford CJ Redman JS Alonso D. Hepatitis C-positive liver transplantation: outcomes and current practice. Curr Opin Organ Transplant. 2021;26:115–120. 33595978 10.1097/MOT.0000000000000848

[5] Goldaracena N Cullen JM Kim DS. Expanding the donor pool for liver transplantation with marginal donors. Int J Surg. 2020;82:30–35. 32422385 10.1016/j.ijsu.2020.05.024

[6] Torabi J Graham JA Choinski K. Young donors with severe acute kidney injury offer an opportunity to expand the donor pool. Am J Surg. 2019;218:7–13. 31003717 10.1016/j.amjsurg.2019.04.005

[7] Elwir S Lake J. Current status of liver allocation in the United States. Gastroenterol Hepatol (N Y). 2016;12:166–170. 27231445 PMC4872844

[8] Glorioso JM. Kidney allocation policy: past, present, and future. Adv Chronic Kidney Dis. 2021;28:511–516. 35367019 10.1053/j.ackd.2022.01.006

[9] Watson CJ Hunt F Messer S. In situ normothermic perfusion of livers in controlled circulatory death donation may prevent ischemic cholangiopathy and improve graft survival. Am J Transplant. 2019;19:1745–1758. 30589499 10.1111/ajt.15241

[10] Mergental H Laing RW Kirkham AJ. Transplantation of discarded livers following viability testing with normothermic machine perfusion. Nat Commun. 2020;11:2939. 32546694 10.1038/s41467-020-16251-3 PMC7298000

[11] Ivanics T Abreu P De Martin E. Changing trends in liver transplantation: challenges and solutions. Transplantation. 2021;105:743–756. 32910093 10.1097/TP.0000000000003454

[12] Lewis A Koukoura A Tsianos GI. Organ donation in the US and Europe: the supply vs demand imbalance. Transplant Rev (Orlando). 2021;35:100585. 33071161 10.1016/j.trre.2020.100585

[13] Müller PC Kabacam G Vibert E. Current status of liver transplantation in Europe. Int J Surg. 2020;82:22–29. 10.1016/j.ijsu.2020.05.062 32454252

[14] Lentine KL Naik AS Schnitzler MA. Variation in use of procurement biopsies and its implications for discard of deceased donor kidneys recovered for transplantation. Am J Transplant. 2019;19:2241–2251. 30809941 10.1111/ajt.15325

[15] Carpenter D Husain SA Brennan C. Procurement biopsies in the evaluation of deceased donor kidneys. Clin J Am Soc Nephrol. 2018;13:1876–1885. 30361336 10.2215/CJN.04150418 PMC6302333

[16] Vodkin I Kuo A. Extended criteria donors in liver transplantation. Clin Liver Dis. 2017;21:289–301. 28364814 10.1016/j.cld.2016.12.004

[17] Nasralla D Coussios CC Mergental H; Consortium for Organ Preservation in Europe. A randomized trial of normothermic preservation in liver transplantation. Nature. 2018;557:50–56. 29670285 10.1038/s41586-018-0047-9

[18] van Rijn R Schurink IJ de Vries Y. Hypothermic machine perfusion in liver transplantation—a randomized trial. N Engl J Med. 2021;384:1391–1401. 33626248 10.1056/NEJMoa2031532

[19] Schlegel A Mueller M Muller X. A multicenter randomized-controlled trial of hypothermic oxygenated perfusion (HOPE) for human liver grafts before transplantation. J Hepatol. 2023;78:783–793. 36681160 10.1016/j.jhep.2022.12.030

[20] Brüggenwirth IM Lantinga VA Rayar M. Prolonged dual hypothermic oxygenated machine preservation (DHOPE-PRO) in liver transplantation: study protocol for a stage 2, prospective, dual-arm, safety and feasibility clinical trial. BMJ Open Gastroenterol. 2022;9:e000842. 10.1136/bmjgast-2021-000842 PMC8764996 35039326

[21] Watson CJ Kosmoliaptsis V Randle LV. Normothermic perfusion in the assessment and preservation of declined livers before transplantation: hyperoxia and vasoplegia—important lessons from the first 12 cases. Transplantation. 2017;101:1084. 28437389 10.1097/TP.0000000000001661 PMC5642347

[22] Dengu F Abbas SH Ebeling G. Normothermic machine perfusion (NMP) of the liver as a platform for therapeutic interventions during ex-vivo liver preservation: a review. J Clin Med. 2020;9:1046. 32272760 10.3390/jcm9041046 PMC7231144

[23] Orman ES Mayorga ME Wheeler SB. Declining liver graft quality threatens the future of liver transplantation in the United States. Liver Transpl. 2015;21:1040–1050. 25939487 10.1002/lt.24160 PMC4566853

[24] Croome KP Taner CB. The changing landscapes in DCD liver transplantation. Curr Transplant Rep. 2020;7:194–204. 32837828 10.1007/s40472-020-00283-1 PMC7357263

